# A Liposomal Formulation to Exploit the Bioactive Potential of an Extract from *Graciano* Grape Pomace

**DOI:** 10.3390/antiox11071270

**Published:** 2022-06-27

**Authors:** Carlos Asensio-Regalado, Rosa María Alonso-Salces, Blanca Gallo, Luis A. Berrueta, Clara Porcedda, Francesca Pintus, Antonio Vassallo, Carla Caddeo

**Affiliations:** 1 Department of Analytical Chemistry, University of the Basque Country UPV/EHU, P.O. Box 644, 48080 Bilbao, Spain; carlos.asensio@ehu.eus (C.A.-R.); blanca.gallo@ehu.eus (B.G.); luisangel.berrueta@ehu.eus (L.A.B.); 2 Consejo Nacional de Investigaciones Científicas y Técnicas (CONICET), CIAS-IIPROSAM, Facultad de Ciencias Exactas y Naturales, Universidad Nacional de Mar del Plata, Funes 3350, Mar de Plata 7600, Argentina; rosamaria.alonsosalces@gmail.com; 3 Department of Biomedical Sciences, University of Cagliari, SS 554-Bivio per Sestu, Monserrato, 09042 Cagliari, Italy; porcedda.clara@gmail.com; 4 Department of Scienze della Vita e dell’Ambiente, Sezione Biomedica, University of Cagliari, SS 554-Bivio per Sestu, 09042 Cagliari, Italy; fpintus@unica.it; 5 Department of Science, University of Basilicata, Viale dell’Ateneo Lucano 10, 85100 Potenza, Italy; 6 Department of Scienze della Vita e dell’Ambiente, Sezione di Scienze del Farmaco, University of Cagliari, Via Ospedale 72, 09124 Cagliari, Italy

**Keywords:** grape pomace, green extraction, liposomes, antioxidant, skin cells, cytocompatibility

## Abstract

Antioxidant compounds with health benefits can be found in food processing residues, such as grape pomace. In this study, antioxidants were identified and quantified in an extract obtained from Graciano red grape pomace via a green process. The antioxidant activity of the extract was assessed by the DPPH and FRAP tests, and the phenolic content by the Folin–Ciocalteu test. Furthermore, nanotechnologies were employed to produce a safe and effective formulation that would exploit the antioxidant potential of the extract for skin applications. Anthocyanins, flavan-3-ols and flavanols were the main constituents of the grape pomace extract. Phospholipid vesicles, namely liposomes, were prepared and characterized. Cryo-TEM images showed that the extract-loaded liposomes were predominantly spherical/elongated, small, unilamellar vesicles. Light scattering results revealed that the liposomes were small (~100 nm), homogeneously dispersed, and stable during storage. The non-toxicity of the liposomal formulation was demonstrated in vitro in skin cells, suggesting its possible safe use. These findings indicate that an extract with antioxidant properties can be obtained from food processing residues, and a liposomal formulation can be developed to exploit its bioactive value, resulting in a promising healthy product.

## 1. Introduction

Phenolic compounds can be naturally found in fruits and plants. Hence, they are thoroughly studied and their antioxidant, antimicrobial and anti-inflammatory properties have been demonstrated [1]. Among fruits, grape is one of the richest sources of phenolic compounds, such as anthocyanins, catechins, procyanidins, and tannins [2], which mostly locate in the skin and seeds [3].

Grape is commonly used for winemaking, and wine consumption has been linked to numerous health benefits [4,5]. After winemaking, a large amount of phenolic compounds still remain in the pomace [6,7]. These compounds can be extracted from the pomace and the extract used for health-related purposes. Therefore, the pomace represents an opportunity as a low-cost source of antioxidants, as well as an ecosustainable alternative to manage food waste.

The extraction step is key in maximizing the health potential of the pomace through the production of an extract as rich as possible in antioxidants. As such, the use of green and simple procedures is preferred [8]. Both the pharmaceutical and cosmetic sectors could benefit from the repurposing of food processing residues [9,10,11]. Indeed, an antioxidant extract might be formulated into a nanoparticle-based product with high potential for application. Over the last years, nanoparticles, among which liposomes are the best-investigated system, have gained wide attention from both industry and academia. Their use offers numerous advantages: nanoparticles can load a single drug or a multi-component extract increasing their solubility and bioavailability, provide protection against degradation, control the release rate, increase efficacy and reduce toxicity [12,13].

In this study, an extract with antioxidant properties was obtained from grape pomace via a simple, green procedure, characterized by liquid chromatography and mass spectrometry, and incorporated into liposomes. The liposomes were studied in terms of morphology, size, charge, entrapment efficiency, stability and cytocompatibility. The aims of the liposomal formulation were (i) to protect the phenolic antioxidants of the extract, which are known to be prone to degradation; (ii) to increase their bioavailability, which is generally poor due to low solubility in water [14]; (iii) to not interfere with the antioxidant activity of the extract; (iv) to allow the development of a safe product that could be applied onto the skin to treat oxidative conditions.

## 2. Materials and Methods

### 2.1. Materials

Phospholipid Lipoid S 75 (S75) was from Lipoid GmbH (Ludwigshafen, Germany). Malvidin-3-*O*-glucoside (Mv-3-*O*-glc) was provided by Extrasynthèse (Genay, France). HPLC-grade acetonitrile (MeCN) and methanol (MeOH) were purchased from Romil Chemical Ltd. (Heidelberg, Germany). Trifluoroacetic acid (TFA), Folin–Ciocalteu’s reagent, 6-hydroxy-2,5,7,8-tetramethylchroman-2-carboxylic acid (Trolox), 2,2-diphenyl-1-picrylhydrazyl (DPPH), 2,4,6-tris(pyridin-2-yl)-1,3,5-triazine (TPTZ) were from Merck/Sigma-Aldrich (Darmstadt, Germany).

### 2.2. Grape Pomace Extract Preparation

Pomace from *Graciano*, an autochthonous red grape cultivar from Spain, was kindly provided by Bodegas Faustino winery (Oyón, Spain). The extraction was carried out according to a previously validated procedure [15]. In short, the pomace was freeze-dried and ground to a powder. Twenty-five g of the powder was dispersed in a 60:40 ethanol:water blend (500 mL), sonicated at r.t., and centrifuged (4 °C, 8000 rpm). The supernatant was collected and analyzed as reported in Section 2.3 and Section 2.4. Thereafter, the supernatant was evaporated under vacuum, freeze-dried and further used for the production of liposomes (see Section 2.5). The percentage yield of the extract was calculated based on the ratio between freeze-dried extract weight and starting dry material weight.

### 2.3. Identification of Phenolic Compounds

The phenolic composition of the *Graciano* extract (GE) was studied by ultra-high performance liquid chromatography and mass spectrometry using an ACQUITY UPLC^TM^ system with a diode array detector and coupled to a quadrupole time of flight mass spectrometer (Waters, Milford, MA, USA). The separation was carried out as described by Asensio-Regalado et al. [15] Briefly, the mobile phases were 0.1% (*v*/*v*) acetic acid in MeOH and 0.1% (*v*/*v*) acetic acid in water delivered at a flow rate of 0.35 mL/min; the volume injected was 5.0 µL; flavan-3-ols, hydroxycinnamic acids, and flavanols were detected at 280, 320, and 370 nm, respectively. Positive and negative ion modes mass spectra were recorded.

### 2.4. Analysis of Anthocyanins

Anthocyanins in GE were identified by high performance liquid chromatography and mass spectrometry using an Alliance 2695 with a diode array detector and coupled to a triple-quadrupole mass spectrometer (Waters) working in positive ion mode. The analysis was carried out as described by Asensio-Regalado et al. [15]. Briefly, the mobile phases were 0.5% (*v*/*v*) TFA in water (A) and MeCN (B) delivered at a flow rate of 0.8 mL/min following a gradient program as previously reported [15]; the volume injected was 50 μL; anthocyanins were detected at 530 nm and their concentrations were expressed as equivalent concentrations of Mv-3-*O*-glc, which was the major anthocyanin in GE.

### 2.5. Vesicle Preparation and Characterization

For the production of liposomes, the freeze-dried *Graciano* extract (GE; 10 mg/mL) and S75 (120 mg/mL) were dispersed in water and subjected to sonication (5 cycles of 5 s on/2 s off + 2 cycles of 3 s on/2 s off) at r.t. using an ultrasound disintegrator (Soniprep 150, MSE Crowley, London, UK). Empty liposomes, that is liposomes without GE, were prepared following the above procedure.

Cryogenic-transmission electron microscopy (cryo-TEM) was used to assess the liposomes’ formation and morphology. The liposomes (3 μL) were loaded on a 300-mesh grid, frozen in liquid ethane using a FEI Vitrobot Mark IV (Eindhoven, The Netherlands), kept below −180 °C in a 626 DH Single Tilt Cryo-Holder (Gatan, France), and transferred to a TECNAI G2 20 TWIN (FEI) working at a 200 kV accelerating voltage in low-dose, bright-field image mode.

Dynamic/electrophoretic light scattering was applied to determine the mean diameter, the polydispersity index (PI) and the zeta potential (ZP) of the liposomes using a Zetasizer nano-ZS (Malvern Panalytical, Worcestershire, UK). The liposomes were diluted with water prior to the analysis.

Dialysis was carried out to remove non-entrapped GE components from the liposomes. GE liposomes (1 mL) were loaded into Spectra/Por^®^ tubing (12–14 kDa molecular weight cutoff; Spectrum Labs, DG Breda, The Netherlands) and gently stirred for 2 h in water (2 L) at r.t.. Non-dialyzed and dialyzed GE liposomes were disrupted with a 40:60 MeOH:water blend (1:50 dilution) and processed as described in Section 2.4 to quantify anthocyanins. The entrapment efficiency (EE) was calculated as a percentage of the anthocyanins quantified in dialyzed vs. non-dialyzed liposomes.

### 2.6. Total Phenolic Content and Antioxidant Assays

The total phenolic content of GE, in a methanol solution and in the liposomes (10 mg/mL), was estimated by the Folin–Ciocalteu assay, with minor modifications [16]. Prior to the test, the liposomes were disrupted by sonication (6 cycles of 10 s on/2 s off) at r.t. to release the GE components. The samples (10 µL) were incubated with Folin–Ciocalteu’s reagent (50 µL) and water (790 µL) for 1 min; 20% aqueous sodium carbonate (150 µL) was added and incubated for 45 min, at room temperature in the dark. The samples were centrifuged (4 °C, 400 rpm), and the absorbance (Abs) of the supernatant was recorded at 750 nm. The total phenolic content was expressed as µg of gallic acid equivalents (GAE)/mL of solution.

The antioxidant activity of GE, in a methanol solution and in the liposomes (10 mg/mL), was estimated by the DPPH assay. The samples (40 µL) were incubated with a 25 µM DPPH methanol solution (2 mL) for 30 min, at room temperature in the dark. The Abs was read at 517 nm. The antioxidant activity (AA) was calculated as a function of the decrease in Abs induced by the samples according to Equation (1):

AA = ((Abs_DPPH_ − Abs_sample_)/Abs_DPPH_) × 100 (1)

The antioxidant activity was also expressed as Trolox Equivalents (TE). The TE values, expressed as µg TE/mL solution, were calculated using a calibration curve (Trolox concentration range: 0–250 µg/mL).

Furthermore, the ferric reducing antioxidant power (FRAP) assay was performed to assess the ability of GE, in a methanol solution and in the liposomes (10 mg/mL), to reduce Fe^3+^-TPTZ to Fe^2+^-TPTZ that induces an increase in absorption [17]. The samples (20 µL) were incubated with a TPTZ-ferric solution (2 mL) for 4 min, at room temperature in the dark. The increase in Abs was recorded at 593 nm. The results, expressed as µg Fe^2+^ equivalents (FE)/mL solution, were calculated using a calibration curve (FeSO_4_ concentration range: 0–1200 µg/mL).

### 2.7. Cell Culture

Human keratinocytes (HaCaT; CLS–Cell Lines Service, Eppelheim, Germany) from passages 15–30 were cultured under standard conditions (5% CO_2_, 95% relative humidity and 37 °C) in Dulbecco’s Modified Eagle’s Medium plus 1% penicillin/streptomycin (Euroclone, Milan, Italy) and 10% fetal bovine serum (Gibco, NY, USA). Cell viability was measured by the 3-(4,5-dimethylthiazol-2-yl)-2,5-diphenyltetrazolium bromide (MTT) assay, as previously reported [18]. Briefly, the skin cells were seeded in 96-well plates (10^4^ cells/well) and exposed for 24 h to GE ethanol solution and GE liposomes, properly diluted to reach the required GE concentrations (0.1, 1 and 10 µg/mL). Empty liposomes were subjected to the same dilutions for an appropriate comparison. Thereafter, the MTT solution was added. After 3 h, dimethyl sulfoxide was added to dissolve the formazan crystals, and the Abs was recorded at 590 nm.

### 2.8. Statistical Analysis

Results are reported as means ± standard deviations (SD). Student’s *t*-test was used to measure the significant differences between groups.

## 3. Results and Discussion

### 3.1. Phenolic Compounds in Graciano Pomace Extract

**Figure 1:**
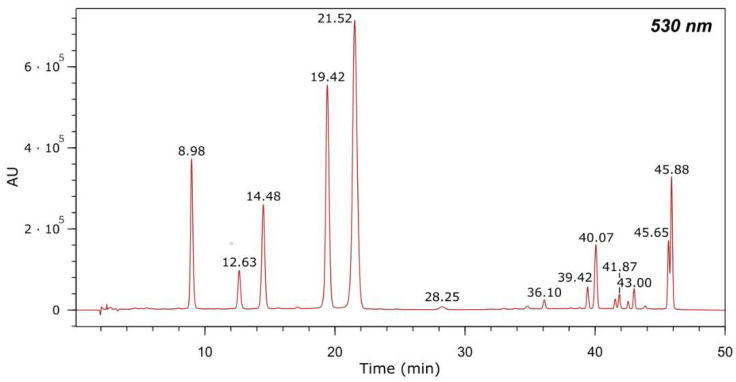
Chromatogram of the anthocyanins in GE detected at 530 nm by HPLC-DAD-MS. Thirteen anthocyanins were identified as reported in Table 1.

After extraction in ethanol/water and freeze-drying, GE was obtained as a purple paste. The yield of the extraction was 42.4%. Based on previously reported data [19], anthocyanins represent the major components of red grape pomaces. For this reason, our study was focused on the quantification of these compounds in GE (Figure 1 and Table 1). In addition, the phenolic composition was determined. Phenolic compounds were identified based on their retention time (t_R_), UV-Vis and MS spectra (Table 2). Ten flavan-3-ols ((−)-epicatechin and (+)-catechin monomers and trimers); 11 flavanols (derivatives of quercetin, kaempferol and isorhamnetin); 1 dihydroflavanol; 1 hydroxycinnamic acid derivative of *p*-coumaric acid; 1 hydroxybenzoic acid; and 13 anthocyanins (3-*O*-glucosides of delphinidin, cyanidin, petunidin, peonidin, and malvidin) were identified in GE. These findings are in line with previous data reporting anthocyanins as the main components of extracts from red grape skin, with Mv-3-*O*-glc being the most abundant [20,21]. Among the other components, phenolic acids, flavan-3-ols and flavanols have been reported as the most representative classes [22]. Flavan-3-ols oligomers and polymers, such as (−)-epicatechin and (+)-catechin, have been found in grape seed extracts, as well [23].

### 3.2. Vesicle Characterization

The aim of this study was to produce an extract from food processing residues by a green process and exploit its bioactive potential by using nanotechnologies. More specifically, the feasibility to produce a safe and effective nanoformulation for a possible application on the skin was evaluated. Liposomes were produced and characterized in terms of size, PI and ZP. To evaluate the impact of GE on the liposomes’ characteristics, the GE liposomes were compared with empty liposomes.

The results presented in Table 3 show that the empty liposomes were approximately 116 nm in diameter, fairly monodispersed (PI 0.25 ± 0.01) and negatively charged (−62 ± 3 mV). The incorporation of GE significantly decreased the mean diameter (*c.a.* 104 nm) and increased the PI (0.29). The ZP values were unaltered. The stability of the liposomal formulations was assessed by measuring these three parameters over two months. No relevant changes were detected. The EE of the liposomes, calculated as a function of the amounts of main anthocyanins (peonidin-3-*O*-(6-*p*-coumaroyl)-glucoside+malvidin-3-*O*-(6-*p*-coumaroyl)-glucoside) in the dialysed vs. non-dialysed liposome dispersions, was 75 ± 30%. Due to the dilution prepared in order to disrupt the vesicles for the analysis of the GE in the liposomes, polyphenolic compounds were found at trace levels. Anthocyanins found in the GE in higher concentrations were still detected, being the major components.

**Figure 2:**
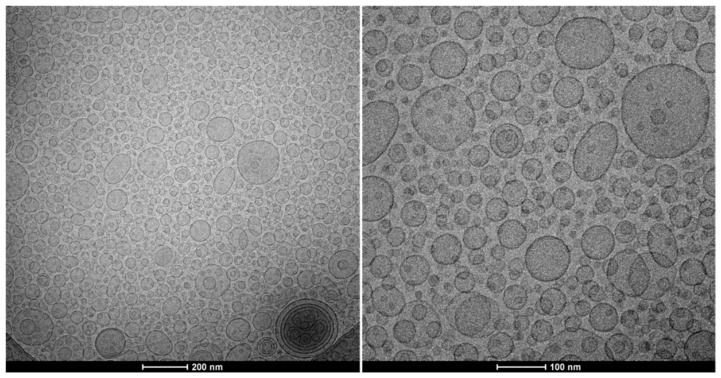
Cryo-TEM images of GE liposomes display spherical/elongated, small, unilamellar vesicles. Two magnifications are shown: 29,000× (**left**) and 62,000× (**right**).

The formation of small liposomes of was corroborated by cryo-TEM images. Figure 2 displays predominantly spherical/elongated, unilamellar vesicles of *c.a.* 100 nm in diameter, which aligns with data from light scattering measurements. Some multivesicular structures were also observed.

### 3.3. Antioxidant Activity of Grape Pomace Extract Liposomes

The AA of GE was determined as radical scavenging ability and ferric reducing ability (Table 4). A GE methanol solution scavenged the DPPH radical (AA 61%; ~200 μg/mL of Trolox equivalents) thanks to the antioxidant compounds present in the extract. Interestingly, the AA of GE liposomes was higher (79%; *p* < 0.01; ~250 μg/mL of Trolox equivalents), due to a contribution from the liposomes’ phospholipids. Indeed, empty liposomes possessed an antioxidant activity themselves (38%, Table 4).

The results of the FRAP assay indicated that the GE liposomes had a strong reducing power (~750 μg FE/mL), which was not statistically different from that of GE methanol solution (Table 4).

The Folin–Ciocalteu assay displayed that the phenolic content of a GE methanol solution was 217 µg GAE/mL (Table 4). The analysis of the GE liposomes gave lower values (191 µg GAE/mL). This is supposed to be due to the fact that this assay does not involve the use of organic solvents and a sonication step was necessary to disrupt the liposomes and free their content. Evidently, this additional process was not as effective as expected. Nevertheless, the overall results show that the formulation in liposomes preserved the antioxidant activity of GE.

### 3.4. Cell Viability

**Figure 3:**
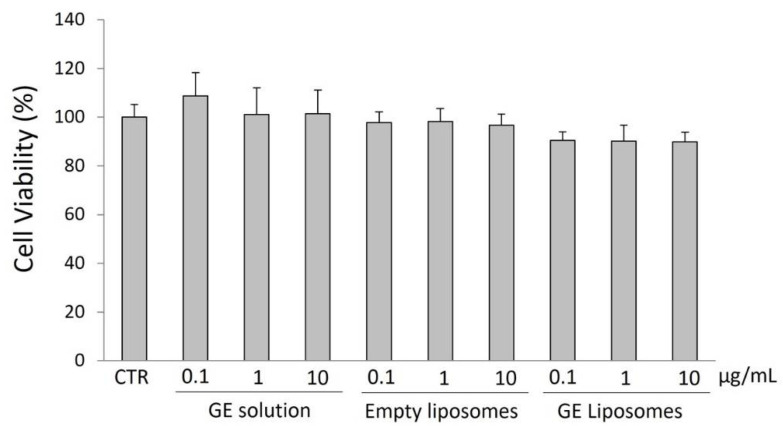
Effect of GE ethanol solution, empty liposomes, and GE liposomes on HaCaT cell viability assayed by the MTT test. The cells were untreated (CTR) or treated with different sample concentrations (0.1–10 μg/mL) for 24 h. The results show no sign of alteration of cell viability.

The effect of free GE and GE liposomes on the viability of HaCaT cells was studied to determine the safety of the extract and the nanoformulation. After 24 h of exposure to the samples (0, 0.1 and 10 μg/mL), the viability of the keratinocytes was not affected. The results reported in Figure 3 show that cell viability was ≥90% for all the tested samples, regardless of the concentration, and without statistically significant differences from untreated cells. This demonstrates that GE and the GE nanoformulation were not cytotoxic.

## 4. Conclusions

The results of this study show that food processing residues, such as grape pomaces, can be used to produce an extract with antioxidant properties via a green process. Furthermore, nanotechnologies were demonstrated to be crucial for developing a formulation complying with standards regarding safety, effectiveness, and usability. Indeed, the proposed liposomal formulation was able to incorporate a grape pomace extract without interfering with its antioxidant properties and was proved to be non-toxic when tested in a cellular system. Therefore, the potential use of the formulation in medical applications holds great promise.

## Figures and Tables

**Table 1:** Quantification of anthocyanins in GE by HPLC-DAD-MS analysis. Retention times (t_R_), mass data and concentration values are presented.

#	Compound	Max. UV-Vis Bands (nm)	t_R_ (min)	[M]^+^ *m/z*	[Y_0_]^+^ *m/z*	Conc. (µg Mv-3-*O*-glc Equivalents/g Freeze-Dried Pomace)
1	Delphinidin-3-*O*-glucoside	276, 525	8.98	465.3	303.2	252.82
2	Cyanidin-3-*O*-glucoside	279, 519	12.63	449.6	287.2	66.05
3	Petunidin-3-*O*-glucoside	276, 525	14.48	479.4	317.0	224.84
4	Peonidin-3-*O*-glucoside	279, 516	19.42	463.1	301.2	517.50
5	Malvidin-3-*O*-glucoside	276, 525	21.52	493.2	331.2	917.45
6	Delphinidin-3-*O*-(6-*O*-acetyl)-glucoside	275, 533	28.25	507.4	303.2	10.26
7	Petunidin-3-*O*-(6-*O*-acetyl)-glucoside	268, 525	36.10	520.9	317.1	12.69
8	Peonidin-3-*O*-(6-*O*-acetyl)-glucoside	279, 525	39.42	505.1	301.3	25.42
9	Malvidin-3-*O*-(6-*O*-acetyl)-glucoside	278, 525	40.07	535.2	331.2	111.41
10	Malvidin-3-*O*-(6-*O*-caffeoyl)-glucoside	280, 530	41.87	655.4	331.1	15.61
11	Petunidin-3-(6-*p*-coumaroyl)-glucoside	279, 532	43.00	625.3	317.1	20.92
12	Peonidin-3-*O*-(6-*p*-coumaroyl)-glucoside ^1^	281, 525	45.65	609.4	301.2	252.19 ^1^
13	Malvidin-3-*O*-(6-*p*-coumaroyl)-glucoside ^1^	281, 532	45.88	639.3	331.2	

^1^ coeluting compounds.

**Table 2:** Identification of phenolic compounds in GE by UHPLC-DAD-MS analysis. Twenty-three compounds were identified including flavan-3-ols, flavanols, dihydroflavanols, hydroxycinnamic acids and hydroxybenzoic acids.

#	Compound	t_R_ (min)	Max. UV-Vis Bands (nm)	Molecular Formula [M + H]^+^	[M + H]^+^ *m/z* Error (mDa)	Molecular Formula [M − H]^−^	[M − H]^−^ *m/z* Error (mDa)
	**Flavan-3-ols**						
1	((Epi)catechin)_3_ (1) ^1^	3.27	283	C_45_H_39_O_18_	867.2144 0.8	C_45_H_37_O_18_	865.1988 0.8
2	Procyanidin B I	5.50	280	C_30_H_27_O_12_	579.1508 0.5	C_30_H_25_O_12_	577.1351 0.5
3	Procyanidin B II	6.42	280	C_30_H_27_O_12_	579.1496 −0.7	C_30_H_25_O_12_	577.1358 1.2
4	((Epi)catechin)3 (2) ^1,2^	7.53	283	C_45_H_39_O_18_	867.2121 −1.5	C_45_H_37_O_18_	865.1988 0.8
5	Catechin ^2^	7.53	278	C_15_H_15_O_6_	291.0873 0.4	C_15_H_13_O_6_	289.0717 0.5
6	Procyanidin B III	8.30	280	C_30_H_27_O_12_	579.1500 −0.3	C_30_H_25_O_12_	577.1349 0.3
7	Procyanidin B IV	12.06	280	C_30_H_27_O_12_	579.1509 0.6	C_30_H_25_O_12_	577.1349 0.3
8	Epicatechin	16.19	278	C_15_H_15_O_6_	291.0869 0.0	C_15_H_13_O_6_	289.0719 0.7
9	Procyanidin B-gallate	19.37	280	C_37_H_31_O_16_	731.1599 −1.3	C_37_H_29_O_16_	729.1398 −5.8
10	((Epi)catechin)_3_ (3) ^1^	20.48	283	C_45_H_39_O_18_	867.2164 2.8	C_45_H_37_O_18_	865.1988 0.8
	**Flavanols**						
11	Quercetin-hexosyl-hexoside-1	20.22	264, 344	C_27_H_31_O_17_	627.1572 1.1	C_27_H_29_O_17_	625.1370 −3.5
12	Quercetin-hexosyl-hexoside-2	25.20	264, 344	C_27_H_31_O_17_	627.1562 0.1	C_27_H_29_O_17_	625.1401 −0.4
13	Quercetin-3-*O*-galactoside	27.58	255, 353	-	n.d. ^3^	C_21_H_19_O_12_	463.0824 −5.3
14	Quercetin-3-*O*-glucuronide	27.85	255, 352	C_21_H_19_O_13_	479.0824 −0.2	C_21_H_17_O_13_	477.0667 −0.2
15	Quercetin-3-*O*-glucoside	28.35	255, 352	-	n.d. ^3^	C_21_H_19_O_12_	463.0918 4.1
16	Kaempferol-3-*O*-galactoside	30.22	265, 345	C_21_H_21_O_11_	449.1081 −0.3	C_21_H_19_O_11_	447.0929 0.2
17	Kaempferol-3-*O*-glucuronide	30.96	265, 345	C_21_H_19_O_12_	463.0878 0.1	C_21_H_17_O_12_	461.0701 −1.9
18	Kaempferol-3-*O*-glucoside	31.49	265, 348	C_21_H_21_O_11_	449.1080 −0.4	C_21_H_19_O_11_	447.0934 0.7
19	Isorhamnetin-3-*O*-galactoside	31.83	254, 352	C_22_H_23_O_12_	479.1192 0.2	C_22_H_21_O_12_	477.1033 0.0
20	Isorhamnetin-3-*O*-glucoside	32.37	254, 352	C_22_H_23_O_12_	479.1188 −0.2	C_22_H_21_O_12_	477.1042 0.9
	**Dihydroflavanols**						
21	Dihydroquercetin-3-*O*-rhamnoside	26.92	255, 352	C_21_H_23_O_11_	451.1241 0.1	C_21_H_21_O_11_	449.1086 0.2
	**Hydroxycinnamic acids**						
22	*p*-coumaroyl hexoside	10.50	313	-	n.d. ^3^	C_15_H_17_O_8_	325.0925 0.2
	**Hydroxybenzoic acids**						
23	Galloyl rhamnoside	3.23	279	-	n.d. ^3^	C_13_H_15_O_9_	315.0717 0.1

^1^ (Epi)catechin: (+)-Catechin or (−)-Epicatechin, unknown isomer. ^2^ coeluting compounds. ^3^ n.d.: not detected.

**Table 3:** Characteristics of GE liposomes in comparison with empty liposomes: mean diameter, PI and ZP measured by light scattering technique. The results show small, homogeneously dispersed, highly negatively charged vesicles, The values are the means ± SD (*n* > 6). ** GE liposomes vs. empty liposomes: ** *p* < 0.01.

Formulation	MD (nm)	PI	ZP (mV)
Empty liposomes	116 ± 7	0.25 ± 0.01	−62 ± 3
GE liposomes	** 104 ± 4	** 0.29 ± 0.01	−65 ± 4

**Table 4:** Antioxidant activity and total phenolic content of GE methanol solution, empty liposomes and GE liposomes. The results of the DPPH, FRAP and Folin–Ciocalteu assays show that the antioxidant activity and the phenolic content of GE were preserved in the liposomal formulation. DPPH values are expressed as AA (%) and as μg TE/mL of solution; FRAP values are expressed as µg FE/mL of solution; total phenolic content is expressed as μg GAE/mL of solution. Mean values ± SDs of at least 3 independent experiments, each performed in triplicate, are reported. ^##^ values statistically different (*p* < 0.01) from GE solution.

Formulation	DPPH Assay		FRAP Assay	Folin–Ciocalteu Assay
	AA (%)	(µg TE/mL)	(µg FE/mL)	(µg GAE/mL)
GE solution	61 ± 3	201 ± 9	819 ± 77	217 ± 10
Empty liposomes	38 ± 3	131 ± 11	339 ± 42	92 ± 9
GE liposomes	*^##^* 79 ± 2	*^##^* 254 ± 9	741 ± 63	*^##^* 191 ± 20

## Data Availability

The data presented in this study are available within this article.
